# A Study of the Antitumour Activity of Four Triorganophosphinegold(I) Thiolates: R_3_ PAu(SR′), R = Ph, Cy, Et; SR′H = 6-Mercaptopurine and R = Et; SR′H = 6-Thioguanine

**DOI:** 10.1155/MBD.1999.361

**Published:** 1999

**Authors:** David Crump, George Siasios, Edward R. T. Tiekink

**Affiliations:** 1 AMRD Operations Pty Ltd 576 Swan Street Richmond Victoria 3121 Australia; 2 Department of Chemistry The University of Adelaide Adelaide 5005 Australia

## Abstract

The antitumour activities of four triorganophosphinegold(I) thiolates, R_2_PAu(S′R) [R = Ph, Cy, Et; SR′H = 6-mercaptopurine and R Et; SR′H = 6-thioguanine] against the National Cancer Institute (NCI) panel of 60 cell lines are reported The [Cy_3_PAu(6-MP)] complex proved to be the more cytotoxic of the four complexes tested. For the 6-MP series, an order of cytotoxicity was established such that the activity followed the order R = Cy > Ph > Et. Sub-panel selectivity against the Leukemia cell lines was found for each of [Cy_3_ PAu(6-MP)] and [Et_3_PAu(6-TG)].


### A STUDY OF THE ANTITUMOUR ACTIVITY OF FOUR TRIORGANOPHOSPHINEGOLD(I) THIOLATES: $R_3PAu(SR')$, R = Ph, Cy, Et; SR'H = 6-MERCAPTOPURINE AND R = Et; SR'H = 6-THIOGUANINE

David Crump[1], George Siasios[2] and Edward R. T. Tiekink[2,*]

[1] AMRAD Operations Pty Ltd, 576 Swan Street, Richmond, Victoria 3121, Australia
[2] Department of Chemistry, The University of Adelaide, Australia 5005

**Abstract**

The antitumour activities of four triorganophosphinegold(I) thiolates, $R_3PAu(S'R)$ [R = Ph, Cy, Et; SR'H = 6-mercaptopurine and R = Et; SR'H = 6-thioguanine] against the National Cancer Institute (NCI) panel of 60 cell lines are reported. The [$Cy_3PAu$(6-MP)] complex proved to be the more cytotoxic of the four complexes tested. For the 6-MP series, an order of cytotoxicity was established such that the activity followed the order R = Cy > Ph > Et. Sub-panel selectivity against the Leukemia cell lines was found for each of [$Cy_3PAu$(6-MP)] and [$Et_3PAu$(6-TG)].

### 1. Introduction

The use of polymeric gold(I) thiolates such as sodium aurothiomalate (Myocrisin) and aurothioglucose (Solganol) in the treatment of rheumatoid arthritis is well documented [1-3]. A monomeric, orally administered species, [(1-thio-β-D-glucopyranose-2,3,4,6-tetraacetato-*S*)(triethylphosphine)gold(I) (Auranofin), is also used clinically in this context. Spurred by the great potential of heavy-metal complexes in the treatment of cancer, *e.g.* cisplatin, it was not surprising that gold(I) complexes such as Auranofin [4] and other phosphinegold(I) thiolates [5,6] were screened for their cytotoxicity. In Adelaide, a series of phosphinegold(I) thiolates, where the thiol is derived from a thio-analogue of a nucleobase or closely related species, have been characterised and both their anti-arthritic activity (*in vivo*) [7-9] and cytotoxicity (*in vitro* and *in vivo*) [10-13] examined. Phosphinegold(I) complexes containing thiolates derived from 6-mercaptopurine (**1**) and 6-thio-guanine (**2**) were generally found to be the most effective in ameliorating the manifestations of induced autoallergic polyarthritis in dark Agouti rats, a gold-sensitive rat strain [14]. Comparisons, *i.e.* activity and toxicity, with the clinically used Myocrisin and Auranofin were favourable, in this model [8,9]. The cytotoxicity of these and related species have also been investigated *in vitro* and in one case preliminary *in vivo* results were obtained [13].

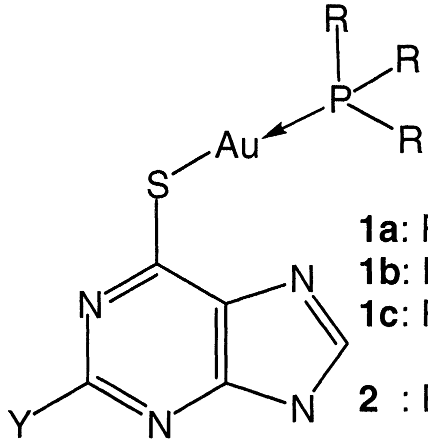

**1a**: R = Ph, Y = H
**1b**: R = Cy, Y = H
**1c**: R = PEt, Y = H

**2** : R = Et, Y = $NH_2$

These studies were encouraging and hence, **1a-c** and **2** were submitted for evaluation to the National Cancer Institute against their panel of cell lines. The results of these screens are presented herein.

* email: etiekink@chemistry.adelaide.edu.au

### 2. Experimental

### 2.1 Synthesis

The complexes were prepared as reported previously [8].

### 2.2. Antitumour screening

The *in vitro* antitumour screening were performed at the Department of Health & Human Services, National Institues of Health, National Cancer Institute, Bethesda, Maryland 20892, USA. A panel of 60 human tumour lines derived from the following nine cancer types: Leukemia, Non-Small Cell Lung Cancer, Colon Cancer, Central Nervous System (CNS) Cancer, Melanoma, Ovarian Cancer, Renal Cancer, Prostate Cancer and Breast Cancer, was employed using the established protocol [15]. The cells lines investigated are

**Leukemia**: CCRF-CEM, HL-60 (TB), K-562, MOLT-4, RPMI-8226, and SR;

**Non-Small Cell Lung Cancer**: A549/ATCC, HOP-62, HOP-92, NCI-H226, NCI-H23, NCI-H322M, and NCI-H522;

**Colon Cancer**: COLO 205, HCT-116, HCT-15, HT29, KM12, and SW-620;

**Central Nervous System Cancer**: SF-268, SF-295, SF-539, SNB-19, SNB-75, and U251;

**Melanoma**: LOX IMVI, MALME-3M, M14, SK-MEL-2, SK-MEL-28, SK-MEL-5, UACC-257, and UACC-62;

**Ovarian Cancer**: IGROV1, OVCAR-3, OVCAR-4, OVCAR-5, OVCAR-8, and SK-OV-3;

**Renal Cancer**: 786-0, A498, ACHN, CAKI-1, RXF 393, SN12C, TK-10, and UO-31;

**Prostate Cancer**: PC-3 and DU-145;

**Breast Cancer**: MCF7, MCF7/ADR-RES, MDA-MB-231/ATCC, HS 578T, MDA-MB-435, MDA-N, BT-549, and T-47D.

### 3. Assays

### 3.1 Mean response concentrations

The effect of administration of the complexes **1a-c** and **2** on the health of the cell lines is represented by the number of viable cells remaining after treatment. This number may be moderated by i) a decrease in cell growth and proliferation, and ii) cell death. Three response parameters are presented to indicate the response of the cells to the gold complexes. These are i) $GI_{50}$, the concentration of gold complex that yields 50 % Growth Inhibition of the cells (*i.e.* percentage growth = +50); ii) TGI, the concentration that causes Total Growth Inhibition (*i.e.* percentage growth = 0); and iii) $LC_{50}$, the concentration of gold complex at which only 50 % of the cells are viable (*i.e.* percentage growth = -50). The results of the screening were analysed at the NCI using the program COMPARE [16]. The graphical results (logarithmic scale) are presented in Figures 1 –4 for **1a-c** and **2**, respectively. The graphs show the relative sensitivities of the cell lines, derived from the $GI_{50}$, TGI and $LC_{50}$ results, to the gold complexes. The bars projecting to the right represent an increase in the sensitivity of the cell line to the gold complex compared with the average sensitivity. Conversely, bars projecting to the left represent a decrease in sensitivity. The mean response concentrations for each of the gold complexes are collected in Table 1.

**Table 1.** Mean response concentrations (M) for the four phosphinegold(I) thiolates screened against the NCI panel of cell lines

| Mean response parameter | [$Ph_3$PAu(6-MP)] | [$Cy_3$PAu(6-MP)] | [$Et_3$PAu(6-MP)] | [$Et_3$PAu(6-TG)] |
|---|---|---|---|---|
| $GI_{50}$ | 1.70e-06 | 1.38e-06 | 1.82e-06 | 1.70e-06 |
| TGI | 4.90e-06 | 4.17e-06 | 6.46e-06 | 7.94e-06 |
| $LC_{50}$ | 1.41e-05 | 1.38e-05 | 2.14e-05 | 2.57e-05 |

**Figure 1.** Mean graph representation of the differential data for [$Ph_3PAu$(6-MP)].

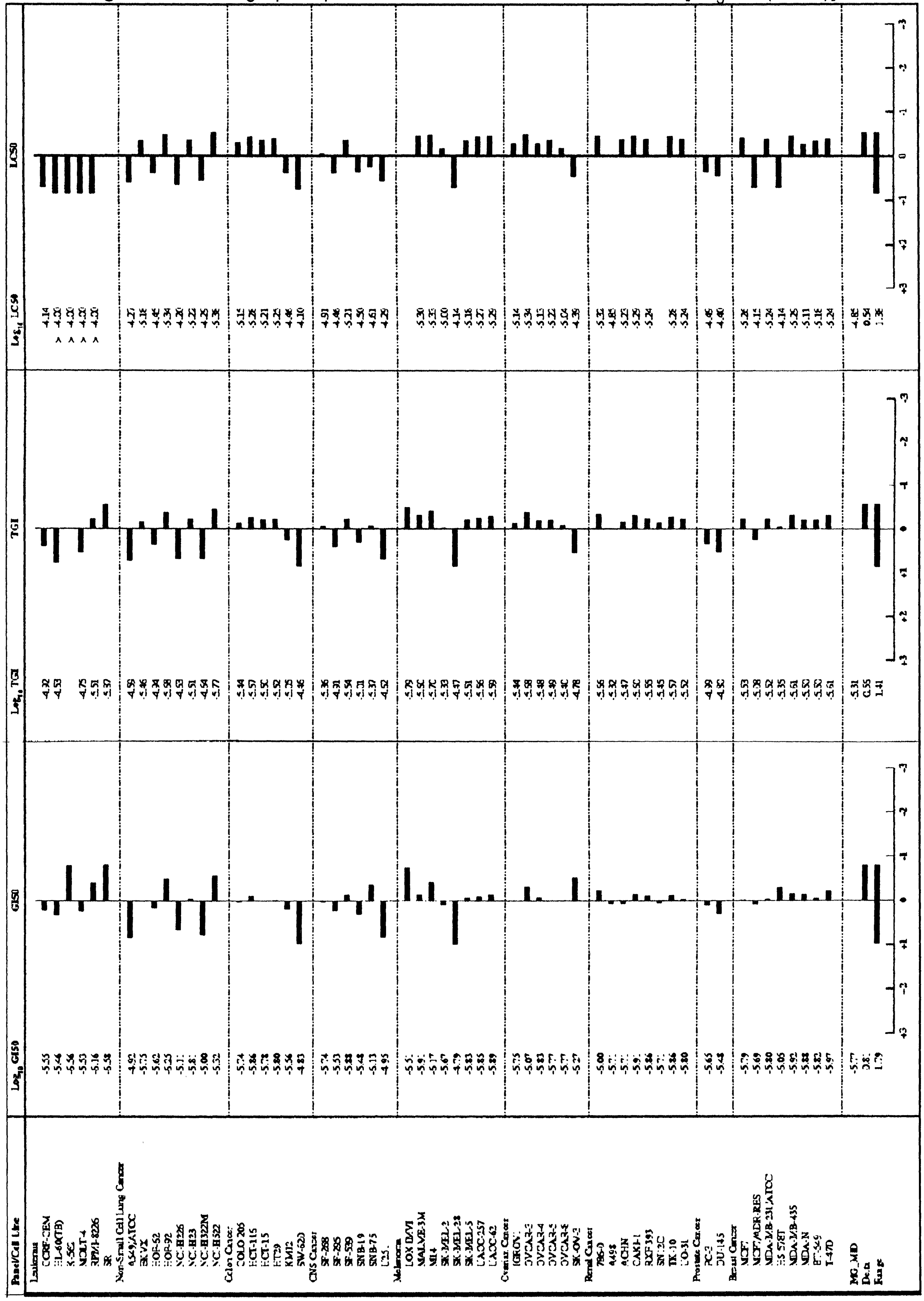

**Figure 2.** Mean graph representation of the differential data for [$Cy_3PAu$(6-MP)].

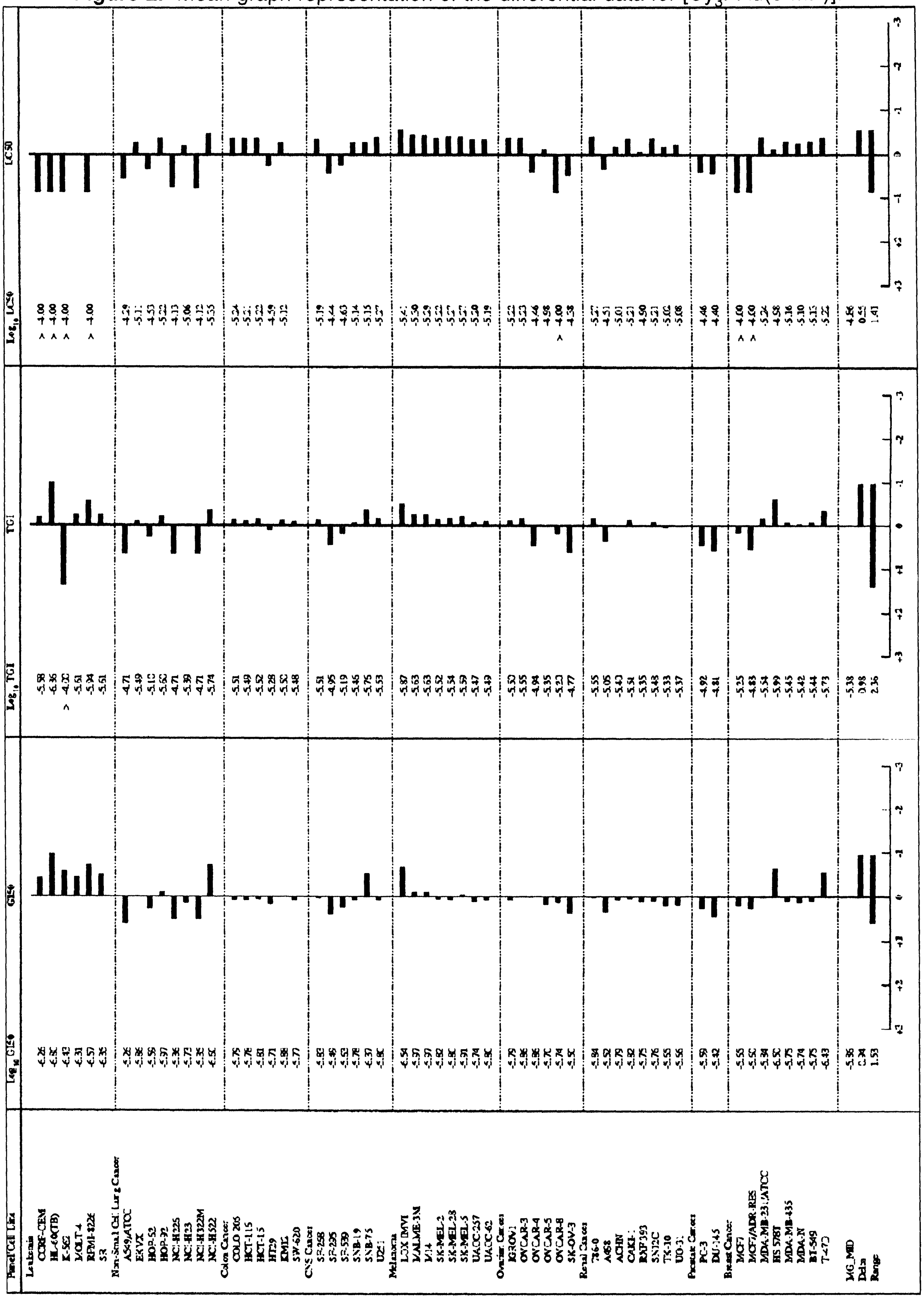

**Figure 3.** Mean graph representation of the differential data for [$Et_3PAu(6\text{-}MP)$].

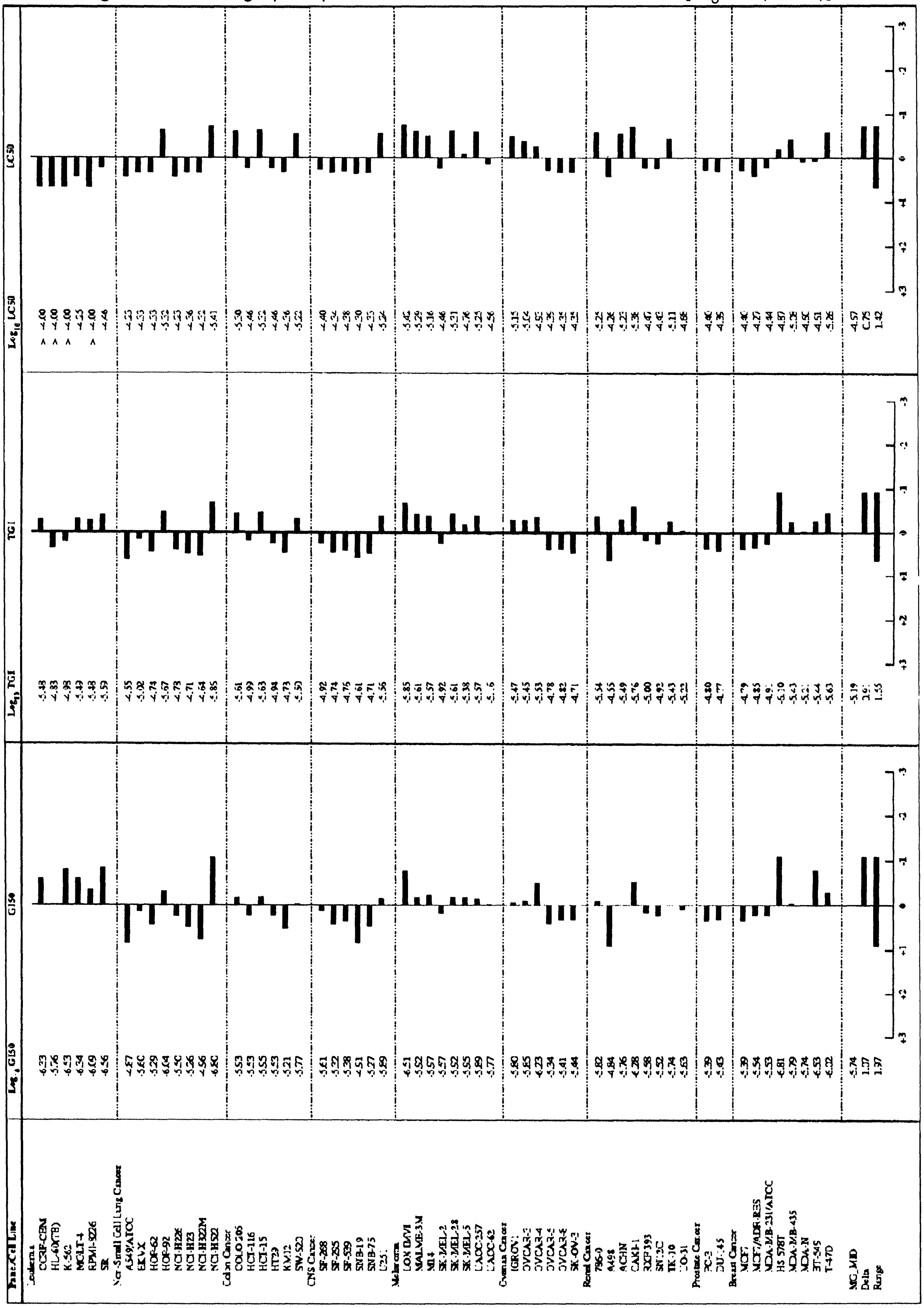

**Figure 4.** Mean graph representation of the differential data for [$Et_3PAu(6\text{-}TG)$].

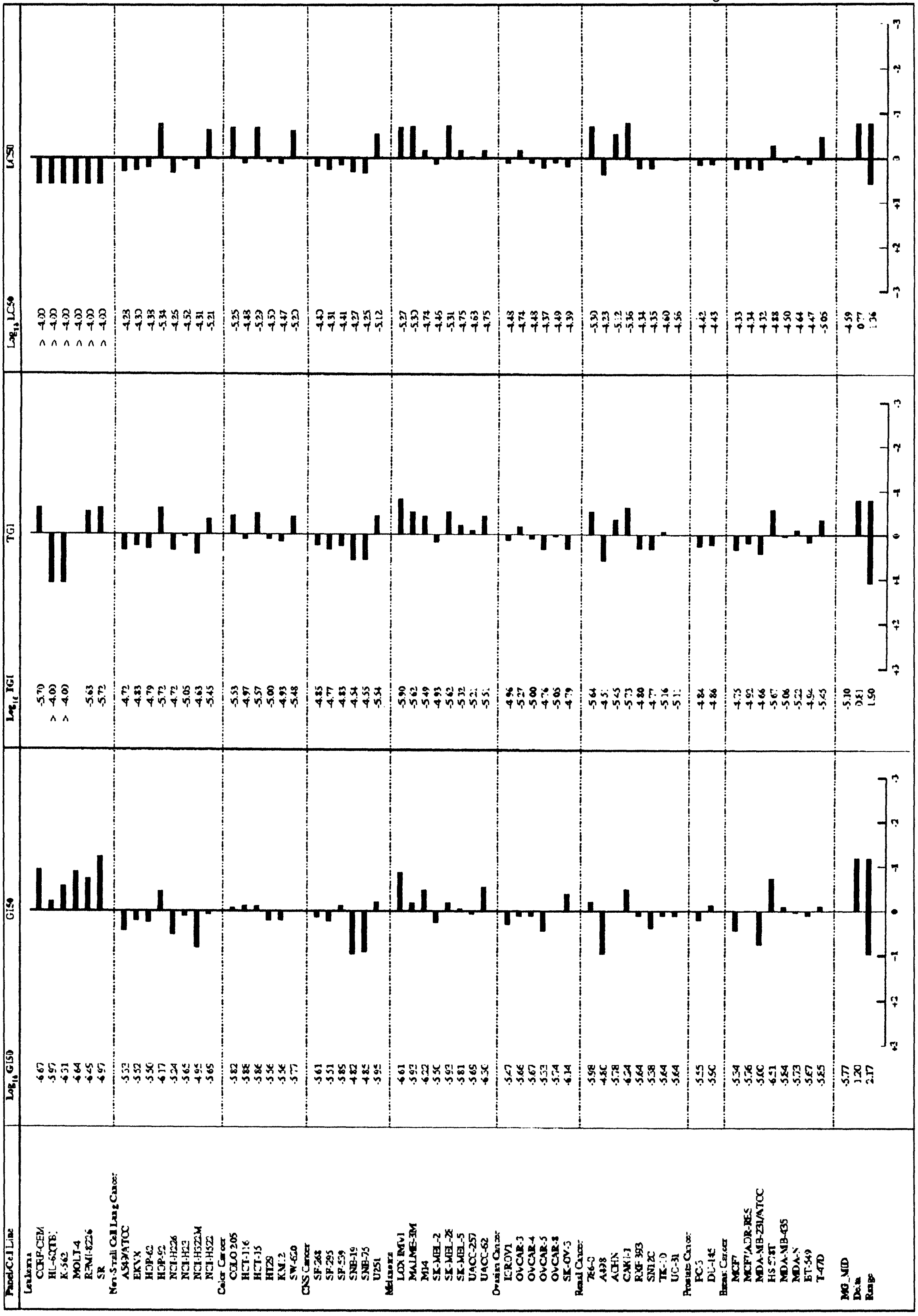

### 3.2 Differential cellular sensitivities

The differential cellular sensitivities for the phosphinegold(I) thiolates are listed in Table 2. The differential sensitivity ($\Delta$) is the mean cytotoxic potency of a particular compound

$$\Delta = \log_{10}(1/X) - \text{mean}[\log_{10}(1/X)];\ X = GI_{50},\ TGI \text{ or } LC_{50}$$

computed over the complete panel of cell lines. Values of lower than 1 represent low sensitivity, values between 1 and 3 indicate moderate sensitivity and those over 3 indicate high sensitivity.

**Table 2.** The differential cellular sensitivities ($\Delta$) for the four phosphinegold(I) thiolates screened against the NCI panel of cell lines

| Differential cellular sensitivity | [$Ph_3PAu$(6-MP)] | [$Cy_3PAu$(6-MP)] | [$Et_3PAu$(6-MP)] | [$Et_3PAu$(6-TG)] |
|---|---|---|---|---|
| $GI_{50}$ | 1.8 | 1.5 | 2.0 | 2.2 |
| TGI | 1.4 | 2.4 | 1.6 | 1.9 |
| $LC_{50}$ | 1.4 | 1.4 | 1.4 | 1.4 |

### 4. Results and discussion

An examination of the mean graph data illustrated in Figure 1 shows that the [$Ph_3PAu$(6-MP)] complex displays no sub-panel selectivity. Maximum cytotoxicity was found against the Leukemia cell lines K-562 and RPMI-8226 as well as the Melanoma cell line LOX IMVI. By contrast, [$Cy_3PAu$(6-MP)] displays sub-panel selectivity, being cytotoxic to the cell lines comprising the Leukemia sub-panel. Maximum cytotoxicity was against the Leukemia line HL-60(TB) with approximately one tenth of the average concentration of the complex was required to achieve the average $GI_{50}$, TGI and $LC_{50}$ values for this particular cell line. For [$Et_3PAu$(6-MP)], with the exception of the the Leukemia cell line HL-60(TB), sub-panel sensitivity was found for the Leukemia sub-panel. Greatest activity was found against HS 578T (Breast Cancer) and NCI-H522 (Non-Small Cell Lung Cancer), each of which display a higher than average response, requiring about one tenth of the concentration to effect the same average responses in $GI_{50}$, TGI and $LC_{50}$. As with [$Cy_3PAu$(6-MP)], [$Et_3PAu$(6-TG)] displays sub-panel selectivity against the Leukemia cell lines. [$Et_3PAu$(6-TG)] also displays maximum activity in this cell line with the SR cell line being the most sensitive.

The four phosphinegold(I) thiolates exhibited moderate responses to all the differential cellular sensitivity parameters as, uniformly, the values for $\Delta$ were less than 3; see in Table 2. The $GI_{50}$, TGI parameters indicate that these complexes inhibited cellular growth at a moderate rate, and, from the $LC_{50}$ values, at best, are moderately cytotoxic.

Of the four phosphinegold(I) thiolate complexes evaluated by the NCI, [$Cy_3PAu$(6-MP)] displayed the greatest cytotoxicity having displayed the lowest average $GI_{50}$, TGI and $LC_{50}$ values. Using this criterion, an order of cytotoxicity may be constructed for the three 6-mercaptopurinate complexes, *i.e.* R = Cy > Ph > Et. The [$Cy_3PAu$(6-MP)], [$Et_3PAu$(6-TG)], and to a lesser extent [$Et_3PAu$(6-MP)], complexes display sub-panel selectivity against the Leukemia cell lines. It is noted that both of the thiopurines, 6-mercaptopurine and 6-thioguanine, have been used in the treatment of Leukemia [17,18] and hence, the activity of their phosphinegold(I) complexes against this cell line is, perhaps, not that surprising. It is also worth noting that previous studies have shown that the presence of the phosphinegold(I) entity imparts greater cytotoxicity compared with the individual thiopurines [11].

Although both of the [$Et_3PAu$(6-MP)] and [$Et_3PAu$(6-TG)] complexes were subjected to repeat screening and subsequently referred to the Biological Evaluation Committee of the NCI, no further action was recommended.

### Acknowledgments

Support from the Australian Research Council is gratefully acknowledged. The authors acknowledge the Developmental Therapeutics Program of the National Cancer Institute, USA for screening. It is with pleasure that the authors acknowledge the advice received from Dr A.B. Mauger, Drug Synthesis & Chemistry Branch (NCI).

**References:**

1. R.V. Parish, *Interdisciplinary Sci. Rev.*, 1992, **17**, 221.
2. R.J. Sue and P.J. Sadler, *Metal-Based Drugs*, 1994, **1**, 107.
3. E.R.T. Tiekink and M.W. Whitehouse, in Handbook of Metal-Ligand Interactions in Biological Fluids. G. Berthon (Ed.) Marcel Dekker, Inc., Vol. 2, 1995, 1266.
4. T.M. Simon, D.H. Kunishima, G.J. Vibert and A. Lorber, *Cancer Res.*, 1985, **45**, 32.
5. C.K. Mirabelli, R.K. Johnson, D.T. Hill, L.F. Faucette, G.R. Girard, G.Y. Kuo, C.-M. Sung, and S.T. Crooke, *J. Med. Chem.*, 1986, **29**, 218.
6. M.P. Arizti, A. Garcia-Orad, F. Sommer, L. Silvestro, P. Massiot, P. Chevallier, J.M. Gutiérrez-Zorrilla, E. Colacio, M. Martinez de Pancorbo and H. Tapiero, *Anticancer Res.*, 1991, **11**, 625.
7. C.S.W. Harker, E.R.T. Tiekink and M.W. Whitehouse, *Inorg. Chim. Acta*, 1991, **181**, 23.
8. P.D. Cookson, E.R.T. Tiekink and M.W. Whitehouse, *Aust. J. Chem.*, 1994, **47**, 577.
9. M.W. Whitehouse, P.D. Cookson, G. Siasios and E.R.T. Tiekink, *Metal-Based Drugs*, 1998, **5**, 245.
10. E.R.T. Tiekink, P.D. Cookson, B.M. Linahan and L.K. Webster, *Metal-Based Drugs*, 1994, **1**, 299
11. L.K. Webster, S. Rainone, E. Horn and E.R.T. Tiekink, *Metal-Based Drugs*, 1996, **3**, 63.
12. D. de Vos, P. Clements, S.M. Pyke, D.R. Smyth and E.R.T. Tiekink, *Metal-Based Drugs*, 1999, **6**, 31.
13. E.R.T. Tiekink, in *Metal Ions in Biology and Medicine*, Ph. Collery, J. Corbella, J.L. Domingo J.C. Etienne and J.M. Llobet (Eds), John Libbey Eurotext, Paris.1996, **4**, 693.
14. I.R. Garett, M.W. Whitehouse, B. Vernon-Roberts and P.M. Brooks, *J. Rheumatol.*, 1985, **12**, 1079.
15. M.R. Boyd, Principles & Practices in Oncology, 1989, **3**(10), 1.
16. K.D. Paull, R.H. Shoemaker, L. Hodes, A. Monks, D.A. Scudiero, L. Rubinstein, J. Plowman, M.R. Boyd, *J. Nat. Cancer Inst.*, 1989, **81**, 1088.
17. J.J. McCormack and D.G. Johns, in *Cancer Chemotherapy: Principles and Practice*, B.A. Chabner and J.M. Collins (Eds) Lippincott Company, Philadelphia, 1990, Chapter 9.
18. J.A. Montgomery, in *Cancer Chemotherapeutic Agents*, W.O. Foye (Ed.) American Chemical Society, Washington, D.C. 1995, Chapter 3, p. 58.

**Received: October 4, 1999 - Accepted: October 12, 1999 -**
**Received in revised camera-ready format: October 14, 1999**

